# Quantum Gravity Spacetime: Universe vs. Multiverse

**DOI:** 10.3390/e27111168

**Published:** 2025-11-19

**Authors:** Massimo Tessarotto, Claudio Cremaschini

**Affiliations:** 1 Department of Mathematics and Geosciences, University of Trieste, Via Valerio 12, 34127 Trieste, Italy; maxtextss@gmail.com; 2 Research Center for Theoretical Physics and Astrophysics, Institute of Physics, Silesian University in Opava, Bezručovo nám.13, CZ-74601 Opava, Czech Republic

**Keywords:** quantum gravity, Heisenberg uncertainty principle, Hamiltonian quantization, 03.50.-z, 04.20.-q, 04.20.Cv, 04.20.Fy, 04.60.-m

## Abstract

Starting from the realization that the theory of quantum gravity (QG) cannot be deterministic due to its intrinsic quantum nature, the requirement is posed that QG should fulfill a suitable Heisenberg Generalized Uncertainty Principle (GUP) to be expressed as a local relationship determined from first principles and expressed in covariant 4-tensor form. We prove that such a principle places also a physical realizability condition denoted as “quantum covariance criterion”, which provides a possible selection rule for physically-admissible spacetimes. Such a requirement is not met by most of current QG theories (e.g., string theory, Geometrodynamics, loop quantum gravity, GUP and minimum-length-theories), which are based on the so-called multiverse representation of space-time in which the variational tensor field coincides with the spacetime metric tensor. However, an alternative is provided by theories characterized by a universe representation, namely in which the variational tensor field differs from the unique “background” metric tensor. It is shown that the latter theories satisfy the said Heisenberg GUP and also fulfill the aforementioned physical realizability condition.

## 1. Introduction: The Problem of Spacetime

The theory of quantum gravity (QG), i.e., the quantization of the Hamiltonian representation of the Einstein field equations (EFEs), is a formidable branch of science, which, however, in some respects must still be regarded apart from mainstream physical literature. This is due to an aspect of current QG theories, which here is called into question: this concerns the type of quantum spacetime usually adopted by some of them. The reason why this happens is already evident in the corresponding classical theory of general relativity (GR) (i.e., before quantization) and can be understood on the basis of the known available routes to achieve a classical Hamiltonian representation for EFE [1]. These are referred to, respectively, as *multiverse* and *universe* (or *background*) *representations* of spacetime (see Ref. [2] and citations therein indicated) in which the spacetime metric tensor is considered either a functionally-prescribed variational tensor

(1) $$g\left(r\right)\equiv \left\{g^{\mu \nu }\left(r\right)\right\}\equiv \left\{g_{\mu \nu }\left(r\right)\right\}$$

(with $g^{\mu \nu }$ and $g_{\mu \nu }$ representing the contra- and co-variant components of each metric tensor and indices μ,ν=0,n with n≥3), namely intrinsically non-unique and hence multiple or, respectively, uniquely prescribed in terms of a so-called “background” metric tensor

(2) $$\hat{g}\left(r\right)\equiv \left\{{\hat{g}}^{\mu \nu }\left(r\right)\right\}\equiv \left\{{\hat{g}}_{\mu \nu }\left(r\right)\right\},$$

which in GR and in case of a four-dimensional spacetime should coincide with the solution of EFE. The same feature also occurs after quantization in the corresponding QG theories, although the canonical quantization methods adopted in the two cases may be substantially different because in multiverse treatments the variational fields are subject to (the orthogonality) constraints, while in the universe representations they are usually treated as unconstrained both at the classical [3] and quantum [4] levels. Thus. in particular, in the universe representation of the QG, the quantum background metric tensor $\hat{g}\left(r\right)$ should be determined by means of a suitable quantum-averaged EFE.

For multiverse theories embedded in a four-dimensional spacetime, this means the variational/quantum metric tensor g(r) must be regarded as non-unique, realizing what are known as so-called many worlds representations of spacetime. Accordingly, there should exist infinite possible quantum spacetimes, each one realized by a different quantum metric field tensor g(r) that belongs to a functional setting of constrained tensor fields.

### 1.1. Criticism Against the Multiverse Representations

Regarding the problem of spacetime, George Ellis has been one of the most vocal critics of multiverse representations of spacetime, which he considers “one of the strangest” proposals suggested so far in physics [5]. His main claim, in fact, is that it should be “impossible to test the validity of multiverse” [6], the reason being that, in his view, there does not exist any possible physical realizability condition able to test the validity of multiverse representations of spacetime. Among others [7,8,9,10,11,12,13,14,15,16], the multiverse representations of spacetime mentioned by Ellis include three possible cases that concern QG theories directly:

1. The first one is represented by string theory, commonly considered as the pre-eminent theory of quantum gravity, a theory characterized by a multidimensional spacetime with dimension ≥4, in which the first four dimensions coincide with those of standard GR, while the higher dimensions are quantum. This represents a multiverse theory, the reason being that its metric tensor should be regarded itself as a quantum variable, i.e., intrinsically non-unique, so that each possible value of the quantum metric tensor should generate a parallel universe. In particular, our Universe should be one of many (possibly infinite) four-dimensional spacetimes (so called `braneworlds’) floating in a higher-dimensional space-time.
2. Second, string-theory-based multiverse models obtained by considering continuous or discrete variations of certain quantum physical parameters that are available in the string theory landscape [6].
3. The third one is realized by most of the conventional QG theories, which, unlike string theory, concern exclusively the case of a four-dimensional spacetime. In this case, the existence of multiverse is based on the assumption that the metric tensor g(r) (of the four-dimensional spacetime) itself is considered either variational or a quantum Lagrangian variable, so that infinite possible realizations of the same metric tensor are allowed. According to this view [17], each parallel universe should correspond to a possible realization of the same metric tensor and consequently a related branch of the quantum wave function. This kind of multiverse model is typical of the so-called ADM Hamiltonian theory (Arnowitt, Deser and Misner [18,19]). More generally, such an assumption is typical of the so-called quantum Geometrodynamics QG theories, such as the Wheeler–deWitt equation [20], the Loop Quantum Gravity (for a review, see [21]) as well as theories that adopt some kind of phenomenological generalized uncertainty principle (GUP) which is non-local and not set in 4-tensor form [22,23,24,25] or minimum length theories [26,27] (see also [28,29,30]).

However, an alternative route to QG theory exists. This is represented by a so-called universe representation of spacetime, which is realized by CQG-theory [3,4]. In this case there is only a unique background quantum spacetime $\hat{g}\left(r\right)\equiv \left\{{\hat{g}}_{\mu \nu }\right\},\left\{{\hat{g}}_{\mu \nu }\right\}$. In particular, in the context of QG, $\hat{g}\left(r\right)$ arises in terms of a quantum expectation value of an ensemble of stochastic tensor fields, while the same background metric tensor determines also the 4-tensor properties of all quantum observables. Remarkably, the latter ones include their corresponding quantum expectation values.

### 1.2. The Search of a Physical Realizability Condition for the Quantum Spacetime

Following George Ellis’ strong criticism of the multiverse representation (according to which the multiverse feature cannot be tested experimentally [6]) it might appear that there is no possible physical criterion capable of distinguishing between multiverse and universe representations of spacetime. However, this conclusion is worthy of further critical analysis. In fact, it is true that QG theories should hopefully satisfy all the basic physical requirements demanded by QG phenomenology, which includes, first of all, the principle of covariance with respect to the group of local point transformations [1]. However, the actual fulfillment of tensor properties in multiverse QG theories poses a serious problem of a physical realizability condition. In fact, in the case of multiverse theories, each of the variational field tensors g(r) should also determine the tensor properties of all quantum observable tensor fields, including in principle, even their corresponding quantum expectation values. Therefore, the question arises whether there exists some sort of physical restriction to be placed on QG theories that can guide us to distinguish between the two categories of QG theories, i.e., either multiverse or universe QG theories.

A first possible criterion of this type might be regarded the so-called principle of objectivity, namely the existence of a classical observable that can be tested experimentally by means of a Gedanken experiment. This is represented by the background metric tensor $\hat{g}\left(r\right)$ together with the requirement that the variational tensor fields g(r) are realized by 4—tensor fields prescribed with respect to the background (i.e., universe) spacetime $\left\{Q^{4},\hat{g}\left(r\right)\right\}$. However, such a criterion does not exhibit, by itself, an obvious and compelling character, so that there is no apparent physical reason “a priori” that might demand its fulfillment.

However, even remaining at the classical level yet another possible solution suggests itself. This is related to the dilemma “*determinism versus indeterminism*”, i.e., the dichotomy concerning the Newton’s deterministic cosmo and its possible non-deterministic generalization (see subsequent Section 2.2). The apparent contradiction, however, is overcome at once by all quantum theories of gravity that satisfy a Heisenberg generalized uncertainty principle (GUP), which is local (i.e., it depends on the 4-position $r\equiv \left\{r^{\mu }\right\}$) and applies to 4-tensor quantum variables (and therefore it is objective in character, i.e., it follows directly from first principles and it is frame independent). In fact, different to the current literature on so-called phenomenological GUP theories (see citations above)—where the same principle is based on phenomenological assumptions and furthermore is non-local and therefore non-4-tensor in character—the Heisenberg GUP considered here is based on first principles, i.e., on an axiomatic formulation of QG theory, and is *local* and expressed in terms of an explicit 4-*tensor representation*. As a consequence, such types of theories should be:

- First, covariant in form, namely in the sense that, because of their 4-tensor representation, such theories should hold with respect to arbitrary GR-frames (frame independence).
- Second, they should be objectively non-deterministic, namely they should satisfy a Heisenberg GUP set in 4-tensor form, and therefore themselves should be frame independent.

Hence, this suggests that a second valid alternative may actually exist for the said physical realizability condition. In this paper we intend to show that such a kind of theory is provided by the so-called covariant QG theory (CQG-theory [3,4]) and by the covariant Heisenberg GUP’s reported previously [31,32], and in particular the requirement that the latter should hold for independent 4-tensor conjugate quantum canonical variables $\left\{g_{\mu \nu },\Pi^{\mu \nu }\right\}$, with $\Pi^{\mu \nu }\left(r\right)$ denoting quantum canonical momentum variables conjugate to the generalized Lagrangian coordinates $g_{\mu \nu }\left(r\right)$. We intend to prove (see Section 6) that such a physical realizability condition requires that for arbitrary 4-tensor observables $A^{\mu \nu }\left(g,r\right)$ each of them admits as a quantum expectation value a 4-tensor of the form

(3) $${\bar{A}}^{\mu \nu }=\langle \psi \left|A^{\mu \nu }\psi \right.\rangle ,$$

such that both ${\bar{A}}^{\mu \nu }$ and the observables $A^{\mu \nu }\left(g,r\right)$ are 4-tensors prescribed with respect to the same quantum spacetime. Here, if $\psi_{a}\left(g\right)$ and $\psi_{b}\left(g\right)$ are two arbitrary scalar fields of $g_{\mu \nu }$, then $\langle \psi_{a}|\psi_{b}\rangle$ denotes a scalar product that takes the form

(4) $$\langle \psi_{a}|\psi_{b}\rangle =\int_{D(g)}d\left(g\left(r\right)\right)\psi_{a}^{*}\left(g\left(r\right)\right)\psi_{b}\left(g\left(r\right)\right),$$

and is defined on the vector space $D\left(g\right)\subseteq R^{10}$ which is spanned by the unconstrained and symmetric tensor field $g_{\mu \nu }$. The consequence is that the QG spacetime should be of the universe type, namely $\left\{Q^{4},\hat{g}\left(r\right)\right\}$, which therefore becomes a mandatory requirement for the validity of a QG theory in which Heisenberg GUP’s in 4-tensor form actually apply.

The aim of this paper is to address these topics, with special emphasis on the following goals:

- *GOAL #1*—The paradox “*determinism vs. indeterminism*” arising in the classical theory of spacetime. As we intend to show here it can be properly solved, i.e., in favour of indeterminism, only in the context of appropriate formulations of QG theory for which the Heisenberg GUP’s hold. This therefore poses the issue of the classification of an arbitrary QG theory (QGT) that is suitable for such a task.
- *GOAL #2*—Current QGT’s can be classified according to their spacetime settings and thus distinguished in multiverse and universe representations of space time.
- *GOAL #3*—The corresponding functional settings needed for the related variational formulation of GR are distinguished by *constrained* or *unconstrained* depending on whether the variational function g(r) is treated as constrained or unconstrained.
- *GOAL #4*—The assumption of validity of the Heisenberg GUP’s in 4-tensor form is introduced. This is shown to require that QGT’s should also fulfill the aforementioned non-trivial *physical realizability condition*, i.e., an explicit 4-tensor constraint on the quantum expectation values of quantum 4-tensors. The latter criterion should therefore be set as a mandatory physical constraint requirement needed for the validity of all QGT’s for which Heisenberg GUP’s hold.
- *GOAL #5*—The physical realizability condition can only apply to universe QG theories. In fact, it is well-known that multiverse GUP theories are not set in 4-tensor form, as required for the validity of the same physical realizability condition.

## 2. The Classical Spacetime of Newton’s Deterministic Cosmo

The early motivations of the present work are historical and related to the notion of classical spacetime due to Newton and Galilei. In fact, such a notion dates back to the early days of Galilei’s relativity principle and Newton’s formulation of his namesake equation for non-relativistic classical mechanics (CM). Set in the language of mathematical physics [33] this means that the so-called *Galileian spacetime structure* (recalled in Appendix A) must hold in the same context. As it is well-known, at the end of the 19th century such a picture was overturned by the discovery of the phenomenon of the constant speed of light in vacuum. This required the complete overhaul of CM, due to Einstein, Lorentz, Hilbert and others, with the formulation of special relativity and, subsequently, of GR. This led, respectively, in the two cases, to the notions of *Minkowski* and *Lorentzian spacetime structures*, holding for classical relativistic point particles (see again Appendix A). Thus, the validity of CM was extended to relativistic particle dynamics, respectively, in special and general relativity.

### 2.1. Newton’s Deterministic Cosmo

However, apart from the structure of the classical spacetime, perhaps the most significant consequence of CM is represented by Newtonian cosmology, i.e., the theory which should govern the dynamics of the classical cosmo, namely the ensemble of all observed bodies, regarded as classical, which belong to the classical spacetime, including also planets, stars, interstellar dust, halos and galaxies. This was identified by Newton with an *N*-body Newtonian system obeying Newton’s equations of motion (or their corresponding relativistic generalizations), a system which therefore is characterized by a unique, i.e., deterministic, time evolution. Such a conclusion led to the notion of deterministic cosmo, a result which holds even if one takes into account possible multiple elastic collisions occurring among its *N* point particles (such collisions in fact can immediately be shown to be deterministic too). Such a property probably represents, together with the discovery of his namesake equation holding in CM, Newton’s most famous achievement. Notice that the same picture, i.e., the determinism of the cosmo, occurs also in special and general relativity and applies even if one takes into account the presence of background EM fields and the possible influence of so-called EM radiation-reaction effects.

### 2.2. The Crisis of the Deterministic Principle: The Paradox “Determinism vs. Indeterminism”

However, despite the great respect of contemporaries toward Newton formulation of CM and Newtonian Cosmology, from the beginning, the concept of a deterministic cosmo appeared dubious. In fact, leaving aside philosophical considerations, possible alternative pictures of classical cosmology can also be devised.

One of them is particularly intriguing since it involves replacing the point particles of the *N*-body Newtonian system with corresponding smooth hard spheres (even possibly with an infinitesimal diameter). In fact this implies that, if multiple collisions among hard spheres occur, then the dynamics of the *N*-body Newtonian system can generally (i.e., except for suitably-prescribed initial conditions) become stochastic, i.e., non-deterministic. Since in practice the replacement of the point particles with smooth hard spheres seems almost irrelevant, such a conclusion appears truly paradoxical. In fact, even if multiple collisions become more unlikely for small radii of the spheres, they are nevertheless still possible if their radius remains non-zero. The implication, however, raises *the paradox of “determinism versus indeterminism”* regarding the classical cosmo, which can be considered either deterministic or stochastic depending on the model adopted for the classical particles (point particles versus smooth hard spheres).

Obviously such a paradox cannot be resolved in the framework of CM. This raises the question of how modern science can help in solving the issue of determinism (or lack of determinism) in the cosmo. However, the same paradox suggests that a crucial role for the particles of the cosmo is played by the interactions occurring at the microscopic level among them, i.e., therefore possibly at the quantum level. A consideration which involves the quantum description of the cosmo, i.e., both for matter and the corresponding spacetime, to be described, respectively, in terms of quantum mechanics (QM) and quantum gravity (QG). On the other hand, QM is non-deterministic thanks to the Heisenberg uncertainty (or indeterminacy) principle and generalized Heisenberg uncertainty inequalities, which represent a fundamental ingredient for ordinary QM. Here we refer the interested reader to [32] for further reference.

Therefore, the actual issue concerns QG. In fact, the really definitive answer regarding the solution of the paradox “determinism versus indeterminism” should come from QG itself.

## 3. The GR and Quantum Spacetime: Multiverse and Universe Representations

The distinction between multiverse and universe (or background) representations in the prescription of the spacetime represents a true frontier divide between the variational theories of GR and QG alike. In both cases, the spacetime is typically identified with a four-dimensional Lorentzian spacetime $\left\{Q^{4},g\right\}$ endowed with signature $\left(-,+,+,+\right)$, with $Q^{4}$ denoting the affine space on $R^{4}$ and g(r) the symmetric metric tensor with signature $\left(-,+,+,+\right)$ represented in terms of the coordinates $r\equiv \left\{r^{\mu }\right\}$. However, the difference amounts to consider also two disparate “geometric” viewpoints, in which in the context of GR (and hence QG too) the construction of covariant dynamical equations in 4-tensor form, in particular continuous Hamilton equations in 4-tensor form, may or may not be possible.

Let us distinguish in detail the two spacetime representations.

### 3.1. The Multiverse Representation

In the multiverse representation, the prescription of spacetime amounts to identify the physical spacetime with $\left\{Q^{4},g\right\}$ in terms of a variational (classical or quantum) metric tensor (1). This means that the 4-tensor g((r) is necessarily constrained being subject to the orthogonality conditions, while remaining undetermined. Hence, the spacetime $\left\{Q^{4},g\right\}$ has necessarily infinite possible realizations (so-called Many Worlds spacetime) corresponding to its possible “parallel” realization, each one characterized by different geometric properties that are determined by the same variational tensor g(r). For definiteness, let us assume that $r\left(s\right)\equiv \left\{r^{\mu }\left(s\right)\right\}$ is a geodetics of the variational metric tensor g(r) which spans the four-dimensional space $R^{4},$ and *s* is the arc-length defined on the same curve. Then, the geometric properties of $\left\{Q^{4},g\right\}$ can be identified with the volume element, the Riemann distance, the covariant derivative along an arbitrary finite-length geodetics of the same of spacetime, together with the Ricci and energy-stress tensors, namely:

(5) $$\left\{\begin{array}{c} d\rho =\sqrt{\left|g(r)\right|}d^{4}r,\\ ds=\sqrt{g_{\mu \nu }\left(r\right)dr^{\mu }dr^{\nu }},\\ \frac{d}{ds}\equiv \frac{dr^{\alpha }}{ds}\nabla_{\alpha }+\frac{\partial }{\partial s},\\ R^{\mu \nu }=R^{\mu \nu }\left(g\left(r\right)\right),\\ T^{\mu \nu }=T^{\mu \nu }\left(g\left(r\right)\right). \end{array}\right.$$

Here, $\frac{d}{ds}$ is the (Lagrangian) covariant s—derivative along a generic geodesic curve $r\left(s\right)\equiv \left\{r^{\mu }\left(s\right)\right\}$ of the tensor field g(r), $\nabla_{\alpha }$ denotes the covariant derivative evaluated in terms of the same variational metric tensor g(r), $\frac{\partial }{\partial s}$ is the partial s—derivative, $\frac{dr^{\alpha }}{ds}\equiv t^{\alpha }$ is the tangent 4-vector to the geodesics $r\left(s\right)\equiv \left\{r^{\mu }\left(s\right)\right\}$ for the metric field tensor g(r) and, finally, $R^{\mu \nu }\left(g\left(r\right)\right)$ and $T^{\mu \nu }\left(g\left(r\right)\right)$ are the Ricci and energy-stress tensors. Notice, however, that in the multiverse representation the covariant derivative of the metric tensor g(r) vanishes identically ($\nabla_{\alpha }g\left(r\right)\equiv 0$) so that

(6) $$\frac{d}{ds}g^{\mu \nu }\left(r\left(s\right)\right)=t^{\alpha }\nabla_{\alpha }g^{\mu \nu }\left(r\right)\equiv 0.$$

Therefore, this means that the multiverse representation $\left\{Q^{4},g\right\}$ may not be suitable for the construction—in the context of GR—of covariant dynamical equations in 4-tensor form (such as continuous Hamilton equations). In fact, if one assumes that in the ensuing Lagrangian representation (obtained considering r=r(s) and allowing also for a possible explicit *s* dependence) the quantum-wave function is a smooth function of the variational metric tensor, i.e., of form ψ(r,s)=ψ(g(r(s)),s), then it follows necessarily:

(7) $$\frac{d}{ds}\psi \left(g\left(r\left(s\right)\right),s\right)\equiv \frac{\partial }{\partial s}\psi \left(g\left(r\left(s\right)\right),s\right),$$

which means that, if ψ does not depend explicitly on *s*, then

(8) $$\frac{d}{ds}\psi \left(g\left(r\left(s\right)\right)\right)\equiv 0,$$

namely its covariant time derivative vanishes identically. In other words, the quantum-wave function, when considered a function of the variational metric tensor g(r(s)) which evolves along a geodetics r(s), cannot describe/represent a dynamical behavior.

### 3.2. The Universe Representation

In the universe representation, instead, the spacetime $\left\{Q^{4},\hat{g}\right\}$ is determined by the metric tensor

(9) $$\hat{g}\left(r\right)\equiv \left\{{\hat{g}}^{\mu \nu }\left(r\right)\right\}\equiv \left\{{\hat{g}}_{\mu \nu }\left(r\right)\right\},$$

referred to as a “background” metric tensor, which in the context of GR is identified with a particular solution of the Einstein field equations. Hence, subject to appropriate boundary conditions, it is necessarily unique. This means that both the same spacetime $\left\{Q^{4},\hat{g}\right\}$ and its geometric properties are uniquely determined, the latter ones being provided by

(10) $$\left\{\begin{array}{c} d\rho =\sqrt{\left|\hat{g}\left(r\right)\right|}d^{4}r,\\ ds=\sqrt{{\hat{g}}_{\mu \nu }\left(r\right)dr^{\mu }dr^{\nu }},\\ \hat{\frac{d}{ds}}\equiv \frac{dr^{\alpha }}{ds}{\hat{\nabla }}_{\alpha }+\frac{\partial }{\partial s},\\ {\hat{R}}^{\mu \nu }=R^{\mu \nu }\left(\hat{g}\left(r\right)\right),\\ {\hat{T}}^{\mu \nu }=T^{\mu \nu }\left(\hat{g}\left(r\right)\right). \end{array}\right.$$

Here, all geometrical quantities are prescribed in terms of the background metric tensor $\hat{g}\left(r\right)$. Thus, $\hat{\frac{d}{ds}}$ is the (Lagrangian) covariant s—derivative along a generic geodesic curve $r\left(s\right)\equiv \left\{r^{\mu }\left(s\right)\right\}$ of the tensor field $\hat{g}\left(r\right)$, ${\hat{\nabla }}_{\alpha }$ denotes the covariant derivative evaluated in terms of the background metric tensor $\hat{g}\left(r\right)$, $\frac{\partial }{\partial s}$ is the partial s—derivative, $\frac{dr^{\alpha }}{ds}\equiv t^{\alpha }$ is the tangent 4-vector to the geodesics $r\left(s\right)\equiv \left\{r^{\mu }\left(s\right)\right\}$ defined with respect to the background metric tensor field $\hat{g}\left(r\right)$. Finally ${\hat{R}}^{\mu \nu }$ and ${\hat{T}}^{\mu \nu }$ are the Ricci and energy-stress tensors, which are both assumed to be extremal, namely evaluated in terms of $\hat{g}\left(r\right)$. A basic consequence is that, if g(r) denotes now an arbitrary variational 4-tensor independent of $\hat{g}\left(r\right)$, then for the quantum wave-function, identified with an arbitrary smooth complex function of ψ(g(r(s)),s), its covariant derivative may be regarded as non-vanishing, i.e.,

(11) $$\hat{\frac{d}{ds}}\psi \left(g\left(r\left(s\right)\right),s\right)\neq 0,$$

because generally $\hat{\frac{d}{ds}}g^{\mu \nu }\left(r\left(s\right)\right)\neq 0$ and $\hat{\frac{d}{ds}}g_{\mu \nu }\left(r\left(s\right)\right)\neq 0$, which implies also that

(12) $$\hat{\frac{d}{ds}}\psi \left(g\left(r\left(s\right)\right)\right)\neq 0.$$

Hence, the quantum wave-function may exhibit a dynamical behavior when the 4-position r≡r(s) moves along the geodetics of $\hat{g}\left(r\right)$. This means that the universe representation of spacetime $\left\{Q^{4},\hat{g}\right\}$ may actually result as suitable for the construction of non-trivial covariant dynamical equations (such as continuous quantum Hamilton equations) that are expressed in 4-tensor form.

Let us analyze in detail the prescriptions of the functional settings for the variational field g(r) in the two cases.

## 4. Constrained and Unconstrained Functional Settings for the Variational Formulation of GR

The relevant issue concerns the variational formulation of GR, i.e., for EFE. For this purpose it is necessary to distinguish between multiverse and universe representations of spacetime while also taking into account the possible occurrence of constraints in the corresponding functional settings.

### 4.1. The Constrained Functional Setting for Multiverse Spacetime Structure

Multiverse theories are based on the four-dimensional Lorentzian spacetime $\left\{Q^{4},g\right\}$, where the metric tensor g(r) is variational, i.e., intrinsically non-unique. In addition, the four-dimensional Ricci tensor $R_{\mu \nu }$ and the energy-stress tensor $T_{\mu \nu }$ are considered unconstrained, in the sense that they are assumed to depend explicitly on the same variational metric tensor g(r). In addition, their tensor indices are raised and lowered, respectively, by the contravariant and covariant components of g(r) (see Equation (1)). The assumption that g(r) is a metric tensor implies that it must fulfill the orthogonality constraints $g^{\lambda \nu }g_{\mu \alpha }=\delta_{\alpha }^{\nu }$. Therefore, denoting by $g_{extr}\left(r\right)$ a suitable extremal metric tensor and δg(r) its (arbitrary) variation, the variational g(r) belongs to the so-called constrained multiverse functional setting

(13) $$\begin{array}{ccc} {\left\{g\right\}}_{C} & \equiv & \left\{\left.g\left(r\right)\equiv g_{extr}\left(r\right)+\delta g\left(r\right)\in C^{2}\left(Q^{4}\right)\right|\right.\\ & & \left.g_{\mu \nu }g^{\mu k}=\delta_{\nu }^{k},\right.\\ & & \left.R_{\mu \nu }=R_{\mu \nu }\left(g\left(r\right)\right),\right.\\ & & \left.\left.T_{\mu \nu }=T_{\mu \nu }\left(g\left(r\right)\right)\right.\right\}. \end{array}$$

### 4.2. ADM Constrained Hamiltonian Representation of GR

Customary multiverse theories on the spacetime $\left\{Q^{4},g\right\}$, known as Geometrodynamics (GMD) theories, are based on the adoption of the ${\left\{g,\pi (r)\right\}}_{C}$ setting together with the ADM constrained variational approach [18,19,34]. This is obtained by implementing a 3 + 1 splitting of spacetime and replacing the metric tensor g(r) with the non 4-tensor ADM variables, i.e., introducing the non 4-tensor mapping (where hereon the Latin indexes vary from 1 to 3)

(14) $$g\left(r\right)\to G_{ADM}\left(g\left(r\right)\right)\equiv \left\{N\left(r\right),N_{a}\left(r\right),h_{ab}\left(r\right)\right\},$$

where in terms of the 4-tensor g(r) a possible non-unique definition of the ADM variables is:

(15) $$\begin{array}{ccc} N(r) & = & c\sqrt{g_{0a}\left(r\right)g^{0a}\left(r\right)-g_{00}\left(r\right)},\\ N_{a}\left(r\right) & = & cg_{0a}\left(r\right)=cg_{a0}\left(r\right),\\ h_{ab}\left(r\right) & = & g_{ab}\left(r\right). \end{array}$$

Here, $h_{ab}\left(r\right)$ identifies the 3 × 3 sub-matrix of the variational 4-tensor g(r), while N(r) and $N_{a}\left(r\right)$ are, respectively, denoted as lapse function and shift 3-vector. Furthermore here $r\equiv r^{\mu }\equiv \left(ct,x\right)$, so that in a general GR-frame the ADM variables depend on coordinate time *t* too. In fact, even assuming that in a suitable GR-frame that ADM variables are indeed time independent, by introducing a boost (i.e., a coordinate transformation that mixes spatial and time coordinates), the transformed ADM variables become generally time-dependent too. Thus the assumption, usually introduced in GMD theories, that ADM variables should depend only on the spatial coordinates x, and therefore that the coordinate time *t* is ignorable, is unjustified.

The first step for determining the ADM Hamiltonian formalism consists in representing in terms of the ADM variables the Einstein–Hilbert action functional

(16) $$\begin{array}{ccc} S(g) & = & \int d^{4}rL\left(g,\Lambda_{c}\right)\\ & = & \int d\left(ct\right)\int d\Sigma L_{ADM}\left(X,{\overset{•}{h}}_{ab},N,N^{a},\Lambda_{c}\right)\\ & \equiv & S_{ADM}\left(X,N,N^{a},\Lambda_{c}\right), \end{array}$$

where dΣ is the surface element on the 3-surface $\Sigma_{t}$ of constant *t*, and $X\equiv \left(h_{ab},\pi^{ab}\right)$ identifies a set of conjugate canonical variables. Thus, the momentum $\pi^{ab}$ conjugate to $h_{ab}$ is defined as

(17) $$\pi^{ab}\equiv \frac{\partial L_{ADM}}{\partial {\overset{•}{h}}_{ab}},$$

being ${\overset{•}{h}}_{ab}$ the partial derivative ${\overset{•}{h}}_{ab}\equiv \partial h_{ab}/\partial \left(ct\right).$ Here the notation is standard [18,19]. Thus, $L(g,\Lambda_{c})=$$\sqrt{-\left|g\right|}\left[g_{\mu \nu }R^{\mu \nu }\left(g\right)-2\Lambda_{c}\right]$ is the E-H Lagrangian density which carries the contribution of the cosmological constant $\Lambda_{c}$, while $R\left(g\right)=g_{\mu \nu }R^{\mu \nu }\left(g\right)$ and $R^{\mu \nu }\left(g\right)$, respectively, identify the Ricci 4-scalar and the Ricci 4-tensor, while the invariant volume element dΩ is

(18) $$d\Omega =d^{4}r\sqrt{-\left|g\right|}=d\left(ct\right)d\Sigma N\sqrt{\left|h\right|},$$

where the ADM Lagrangian density $L_{ADM}\left(X,{\overset{•}{h}}_{ab},N,N^{a},\Lambda_{c}\right)$ expressed in terms of the conjugate canonical variables $\left(h_{ab},\pi^{ab}\right)$ reads

(19) $$\begin{array}{ccc} & & \left.L_{ADM}\left(X,{\overset{•}{h}}_{ab},N,N^{a},\Lambda_{c}\right)\equiv \pi^{ab}{\overset{•}{h}}_{ab}-H_{ADM}\left(X,N,N^{a},\Lambda_{c}\right),\right. \end{array}$$

(20) $$\begin{array}{ccc} & & \left.H_{ADM}\left(X,N,N^{a},\Lambda_{c}\right)=NH_{0}-N_{a}H^{a}.\right. \end{array}$$

Here, $H_{0}$ and $H^{a}$ identify the phase functions (“Hamiltonians”)

(21) $$\begin{array}{ccc} H_{0}\left(X,N,N^{a},\Lambda_{c}\right) & = & \sqrt{\left|h\right|}\left[{-}^{\left(3\right)}R\left(h\right)-2\Lambda_{c}+{\left|h\right|}^{-1}\pi^{ab}\pi_{ab}-\frac{1}{2}{\left|h\right|}^{-1}\pi^{2}\right], \end{array}$$

(22) $$\begin{array}{ccc} H^{a}\left(X\right) & = & -2D_{b}\pi^{ba}. \end{array}$$

Finally, R(3)(h)≡hlm(3)Rlm, while Rlm(3)(h) and $D_{b}$ are, respectively, the three-dimensional Ricci tensor and the three-dimensional covariant derivative. The first one is given in terms of the Christoffel symbols $\gamma_{jk}^{i}$ by

(23) Rlm(3)(h)=Rlkmk(3)(h)=∂kγlmk−∂mγklk+γknkγlmn−γmnkγkln,

(24) $$\begin{array}{ccc} \gamma_{jk}^{i} & = & \frac{1}{2}h^{il}\left(\partial_{k}h_{kl}+\partial_{k}h_{jl}-\partial_{l}h_{jk}\right), \end{array}$$

while $D_{b}$ is defined in terms of the four-dimensional covariant derivative $\nabla_{f}$ so that

(25) $$D_{c}\pi_{b}^{a}\equiv h_{d}^{a}h_{b}^{e}h_{c}^{f}\nabla_{f}\pi_{e}^{d}.$$

In order to construct the ADM variational principle we represent the constrained functional setting ${\left\{g\right\}}_{C}$ given above (see Equation (13)) in the ADM variables $h_{ab},{\overset{•}{h}}_{ab},\pi^{ab},N,N^{a}$. This yields the modified constrained functional setting

(26) $$\begin{array}{ccc} & & \left.{\left\{X,N,N^{a}\right\}}_{C}\equiv \right.\\ & & \left\{\left.X\left(r\right)\equiv X_{extr}\left(r\right)+\delta X\left(r\right)\in C^{2}\left(Q^{4}\right)\right|\right.\\ & & \left.N\left(r\right)\equiv N_{extr}\left(r\right)+\delta N\left(r\right)\in C^{2}\left(Q^{4}\right)\right.\\ & & \left.N^{a}\left(r\right)\equiv N_{extr}^{a}\left(r\right)+\delta N^{a}\left(r\right)\in C^{2}\left(Q^{4}\right)\right.\\ & & \left.g_{\mu \nu }g^{\mu k}=\delta_{\nu }^{k},\right.\\ & & \left.R_{\mu \nu }=R_{\mu \nu }\left(h_{ab},N,N^{a}\right),\right.\\ & & \left.\left.T_{\mu \nu }=T_{\mu \nu }\left(h_{ab},N,N^{a}\right)\right.\right\}. \end{array}$$

Then, let us denote by $\delta S_{ADMN}\left(X,{\overset{•}{h}}_{ab}N,N^{a},\Lambda_{c}\right)$ the Frechet derivative

(27) $$\delta S\left(X,\overset{•}{h},N,N^{a},\Lambda_{c}\right)\equiv \lim_{\alpha \to 0}\int dt\int d\Sigma \frac{d}{d\alpha }L_{ADM},$$

where

(28) $$\left\{\begin{array}{cc} h_{ab}= & h_{extrab}+\alpha \delta h_{ab},\\ \pi_{ab}= & \pi_{extrab}+\alpha \delta \pi_{ab},\\ N= & N_{extr}+\alpha \delta N,\\ N^{a}= & N_{extr}^{a}+\alpha \delta N^{a}. \end{array}\right.$$

The variational action principle, required to hold for arbitrary variations $\delta h_{ab},\delta \pi_{ab},\delta N$ and $\delta N^{a}$ belonging to the constrained setting (26), namely

(29) $$\delta S(h_{ab},{\overset{•}{h}}_{ab},\pi_{sb},N,N^{a},\Lambda_{c})=0$$

yields, ignoring boundary contributions, and by identifying the ADM Lagrangian and Hamiltonian densities in terms of Equations (19) and (20). Then, the Euler–Lagrange equations are expressed in terms of the variational derivatives of the Hamiltonian density, i.e.,:

(30) $$\left\{\begin{array}{cc} {\overset{•}{h}}_{ab}= & \frac{\delta H}{\delta \pi^{ab}}=N\frac{\delta H_{0}}{\delta \pi^{ab}}-N_{c}\frac{\delta H^{c}}{\delta \pi^{ab}},\\ {\overset{•}{\pi }}^{ab}= & -\frac{\delta H}{\delta h_{ab}}=-N\frac{\delta H_{0}}{\delta h_{ab}}+N_{c}\frac{\delta H^{c}}{\delta h_{ab}},\\ \;\;\;H_{0}=0, & \\ \;\;\;H^{a}=0. \end{array}\right.$$

In particular, in the second equation one obtains, upon explicit evaluation of the variational derivative and neglect of boundary terms (coming from the variational derivative of the three-dimensional Ricci tensor R(3) [18,19]):

(31) δH0δhab≡∂H0∂hab=−NhRab(3)(h)−12hab(3)Rab(h)++habΛc+Nhab2hπlmπlm−12π2−2Nhπacπcb−12ππab+hDaDbN−habDcDcN++hDcNchabh−2πcaDcNb.

Therefore, due to the dependences in terms of partial spatial derivatives of $h_{ab}$ contained in the three-dimensional Ricci tensor Rab(3)(h), the rhs of Equation (31) generally depends on the first and second order partial derivatives of the three-dimensional metric tensor $h^{lm}$. As a consequence the Euler–Lagrange Equation (30) become intrinsically partial differential equations, which means they cannot be interpreted as Hamiltonian ordinary differential equations, unless the further unphysical constraint condition Rab(3)(h) = const is invoked.

In conclusion, this means that a number of implications follow. Precisely:

1. Equation (31) proves that the identification of the Euler–Lagrange Equation (30) in terms of Hamilton equations is generally not possible. This result is consistent with Ref. [1]. As a consequence, standard canonical quantization methods cannot be generally adopted unless an additional constraint condition is invoked, which permits to recover ordinary differential equations in Hamiltonian form. This requires, besides the validity of the modified constrained functional setting (26), to impose
  (32) Rab(3)=const.,
  where Rab(3) is the three-dimensional Ricci tensor (with indices a,b=1,3). Such a constraint condition, however, remains physically dubious.
2. Unless the variational 4-tensor g(r) is identified with the background spacetime $\hat{g}\left(r\right)$, the theory remains of the multiverse type, in which the metric tensor field, involved in the prescriptions of all quantum observables, is considered variational (and hence it does not generally coincide with the background metric tensor defined above (2)).
3. Provided the ADM variables are assumed independent of the coordinate time *t*, Geometrodynamics theories become time-independent too (i.e., they do not depend explicitly on *t*). Such a property, however, is frame-dependent since it is manifestly violated if a relativistic boost coordinate transformation that mixes space and time coordinates is performed [32]. Hence, such theories are necessarily frame dependent.
4. Since ADM variables (15) are not set in 4-tensor covariant form, all Geometrodynamics QG theories are not realized in 4-tensor form.
5. The quantum expectation values of tensor quantum observables do not generally satisfy 4-tensor transformation properties and therefore the ADM quantum theory cannot generally satisfy an Heisenberg GUP set in the 4-tensor form.

### 4.3. Unconstrained Functional Settings for the Universe Spacetime Structure

In the case of the universe QG spacetime structure, in principle both unconstrained and constrained functional settings can be adopted. For the purpose of the subsequent discussion, however, here it is sufficient to consider only the first case. The universe (or background) spacetime structure is provided by the set $\left\{Q^{4},\hat{g}\right\}$, with $\hat{g}\left(r\right)$ denoting a background symmetric quantum metric tensor. This is prescribed by QG theory in terms of a quantum expectation value of the type

(33) $${\hat{g}}^{\mu \nu }\left(r\right)=\langle \psi \left(r\right)\left|\Delta g^{\mu \nu }\left(r\right)\psi \left(r\right)\right.\rangle .$$

Here, $\langle \psi_{a}|\psi_{b}\rangle$ is an appropriate scalar product to be defined on the QG Hilbert space, while $\Delta g^{\mu \nu }$ denotes a stochastic “displacement” 4-tensor such that

(34) $$\begin{array}{ccc} G^{\mu \nu }\left(r\right) & = & g^{\mu \nu }\left(r\right)-\Delta g^{\mu \nu }\left(r\right), \end{array}$$

(35) $$\begin{array}{ccc} \frac{d}{ds}\Delta g^{\mu \nu }\left(r\right) & = & 0, \end{array}$$

where the last Equation (35) is assumed to hold identically. Here the notations are as follows. First, in Equation (35) $\frac{d}{ds}$ identifies again the covariant s—derivative operator according to the geometric properties prescribed for $\left\{Q^{4},\hat{g}\right\}$ (see Equation (10)), where for simplicity hereon we shall denote as $\frac{d}{ds}$$\equiv \hat{\frac{d}{ds}}$. Furthermore, both $g^{\mu \nu }\left(r\right)$ and $G^{\mu \nu }\left(r\right)$ are 4-tensor fields that denote, respectively, an arbitrary quantum variational field and a suitable “generalized Lagrangian path” (GLP) [32]. Second, the last Equation (35) presupposes the introduction of a parametrization in terms of the arc length (or proper time) *s* (s—parametrization) along a non-null geodetics of the background spacetime $\left\{Q^{4},\hat{g}\right\}$ (by assumption belonging to a family of such geodetics, defined so that any 4-position *r* belongs to a unique geodetics and nearby 4-positions are smoothly dependent on *s*). Thus, if for definiteness r=r(s) is the s—parametrization of the 4-position r=r(s), i.e., *s* labels the 4-position along a non-null geodetics, with *s* being its arc length, hereon we shall denote by $g^{\mu \nu }\left(s\right)\equiv g^{\mu \nu }\left(r\left(s\right)\right)$ and $G^{\mu \nu }\left(s\right)\equiv G^{\mu \nu }\left(r\left(s\right)\right)$ the corresponding parametrizations of the tensor fields $g^{\mu \nu }\left(r\right)$ and $G^{\mu \nu }\left(r\right)$.

More generally, however, in the universe case the metric tensor of the spacetime must be considered as prescribed in terms of an extremal metric tensor $g_{extr}\left(r\right)$. The precise choice of $g_{extr}\left(r\right)$ depends on the kind of variational principle that is adopted (i.e., associated with EFE). Thus, for example in the case that the extremal Euler–Lagrange equation is required to coincide with EFE, then

(36) $$g_{extr}\left(r\right)\equiv \hat{g}\left(r\right),$$

where in classical GR $\hat{g}\left(r\right)$ denotes the background tensor field solution of EFE. Instead, in QG one can show that the same tensor field can be identified in terms of a quantum expectation value of the type (33) [1].

In particular, the Lagrangian 4-tensor field g(r) can always be considered unconstrained [3,4]. In this case, the Lagrangian 4-tensor g(r) is treated as unconstrained so that it belongs to the functional setting

(37) $$\begin{array}{ccc} {\left\{g\right\}}_{U} & \equiv & \left\{\left.g\left(r\right)\equiv g_{extr}\left(r\right)+\delta g\left(r\right)\in C^{2}\left(Q^{4}\right)\right|\right.\\ & & R_{\mu \nu }={\hat{R}}_{\mu \nu }\equiv R_{\mu \nu }\left(\hat{g}\left(r\right)\right),\\ & & \left.T_{\mu \nu }={\hat{T}}_{\mu \nu }\equiv T_{\mu \nu }\left(\hat{g}\left(r\right)\right),\right.\\ & & \left.g^{\mu \nu }={\hat{g}}^{\mu \alpha }{\hat{g}}^{\nu \beta }g_{\alpha \beta }\left(r\right),{\hat{g}}_{\mu \nu }{\hat{g}}^{\mu k}=\delta_{\nu }^{k}\right\}, \end{array}$$

where $g_{extr}\left(r\right)$ is an extremal symmetric metric tensor not necessarily coinciding with $\hat{g}\left(r\right)$. Here we notice that, differently to the multiverse setting, by assumption both the Ricci tensor $R_{\mu \nu }$ and the energy-stress tensor $T_{\mu \nu }$ are considered prescribed, i.e., functions of the background metric tensor $\hat{g}\left(r\right)$.

For completeness we mention here also the corresponding Hamiltonian functional setting. This requires introducing 4-tensor canonical momenta $\pi \left(s\right)\equiv \left\{\pi^{\mu \nu }\left(s\right)\right\}$ (see next Section 4.4). Thus, denoting $x_{Rextr}\left(r\right)\equiv \left\{g_{extr}\left(r\right),\pi_{extr}\left(r\right)\right\}$ the extremal state, with $g_{extr}\left(r\right)$ and $\pi_{extr}\left(r\right)$ to be prescribed, the functional setting for the unconstrained variational Hamiltonian state $x_{R}\left(r\right)\equiv \left\{g(r),\pi (r)\right\}$ reads as follows:

(38) $$\begin{array}{ccc} {\left\{g,\pi \right\}}_{U} & \equiv & \left\{x_{R}\left(r\right)\equiv x_{Rextr}\left(r\right)+\delta x_{R}\left(r\right)\in C^{2}\left(Q^{4}\right)\right.\\ & & \left.R_{\mu \nu }={\hat{R}}_{\mu \nu }\equiv R_{\mu \nu }\left(\hat{g}\left(r\right)\right)\right.\\ & & \left.T_{\mu \nu }={\hat{T}}_{\mu \nu }\equiv T_{\mu \nu }\left(\hat{g}\left(r\right)\right)\right.\\ & & \left.g^{\mu \nu }={\hat{g}}^{\mu \alpha }{\hat{g}}^{\nu \beta }g_{\alpha \beta }\left(r\right),{\hat{g}}_{\mu \nu }{\hat{g}}^{\mu k}=\delta_{\nu }^{k}\right\}. \end{array}$$

### 4.4. Universe Spacetime: Classical and Quantum Covariant Hamiltonian Structures of GR

In this section the covariant Hamiltonian structures of GR, both classical [3] and quantum ones [4], are recalled, including the determination of the background metric tensor $\hat{g}\left(r\right)$ to be determined in terms of the quantum expectation value of the quantum wave-function (33).

Regarding the classical Hamiltonian structure, which holds in the case of the universe spacetime structure, this is a mandatory requirement of covariant classical gravity (CCG) theory (see Ref. [3]). Such a Hamiltonian structure is based on the unconstrained functional setting ${\left\{g,\pi \right\}}_{U}$ indicated above (see Equation (38)). The structure, which can be readily determined in terms of a variational principle, yields the classical Hamiltonian structure of GR, i.e., the abstract Hamiltonian system associated to EFE. For the interested reader the procedure is described in detail in Ref. [3]. The resulting covariant Hamiltonian structure of GR is represented by the set $\left\{x_{R},H_{R}\right\}$, formed by the s—parametrized canonical state $x_{R}\left(s\right)=\left\{g(s),\pi (s)\right\}$, with $g\left(s\right)=\left\{g_{\mu \nu }\left(s\right)\right\}$ and $\pi \left(s\right)\equiv \left\{\pi^{\mu \nu }\left(s\right)\right\}$ both represented by second order 4-tensors. Here, in terms of the “generalized velocities”

(39) $${\overset{•}{g}}_{\mu \nu }\left(s\right)\equiv \frac{d}{ds}g_{\mu \nu }\left(s\right),$$

the momenta $\pi^{\mu \nu }\left(s\right)$ are defined as

(40) $$\pi^{\mu \nu }\left(s\right)=\alpha L\frac{\partial L_{R}}{\partial {\overset{•}{g}}_{\mu \nu }\left(s\right)},$$

with $L_{R}\left(g\left(s\right),\hat{g}\left(r\right),{\overset{•}{g}}_{\mu \nu }\left(s\right)\right)$ and $H_{R}\left(g\left(s\right),\pi \left(s\right),\hat{g}\left(r\right)\right)=$${\overset{•}{g}}_{\mu \nu }\left(s\right)\pi^{\mu \nu }\left(s\right)-L_{R}\left(g\left(s\right),{\overset{•}{g}}_{\mu \nu }\left(s\right),\hat{g}\left(r\right)\right)$ being suitable 4-scalar Lagrangian and Legendre-conjugate Hamiltonian densities, all quantities being defined with respect to the same background spacetime $\left\{Q^{4},\hat{g}\left(r\right)\right\}$. Accordingly, the canonical state $x_{R}\left(s\right)$ obeys the continuous Hamilton equations (i.e., ordinary differential equations)

(41) $$\begin{array}{ccc} \frac{d}{ds}g_{\mu \nu }\left(s\right) & = & \left[g_{\mu \nu },H_{R}\right]=\frac{\partial H_{R}}{\partial \pi^{\mu \nu }}, \end{array}$$

(42) $$\begin{array}{ccc} \frac{d}{ds}\pi^{\mu \nu }\left(s\right) & = & \left[\pi_{\mu \nu },H_{R}\right]=\frac{\partial H_{R}}{\partial g_{\mu \nu }}, \end{array}$$

which satisfy the initial condition

(43) $$x_{R}\left(s_{o}\right)=\left\{g\left(s_{o}\right),\pi \left(s_{o}\right)\right\}.$$

Here, $\left[A,H_{R}\right]$ denotes the Poisson brackets $\left[A,H_{R}\right]\equiv \frac{\partial A}{\partial g^{\mu \nu }}\frac{\partial H_{R}}{\partial \pi_{\mu \nu }}-\frac{\partial A}{\partial \pi_{\mu \nu }}\frac{\partial H_{R}}{\partial g^{\mu \nu }}$, while $\frac{d}{ds}$ is again the covariant s—derivative operator defined above. The Lagrangian and Hamiltonian densities $L_{R}$ and $H_{R}$ take the form

(44) $$\begin{array}{ccc} L_{R} & = & T_{R}-V\left(g,\hat{g},r\left(s\right),s\right), \end{array}$$

(45) $$\begin{array}{ccc} H_{R} & = & T_{R}+V\left(g,\hat{g},r\left(s\right),s\right), \end{array}$$

(46) $$\begin{array}{ccc} V & = & V_{0}+V_{F}, \end{array}$$

where $T_{R}$ and $V(g,\hat{g},r\left(s\right),s)$ are effective kinetic and normalized potential energies, with $V_{o}$ and $V_{F}$ being the vacuum and external contributions. In particular,

(47) $$\begin{array}{ccc} & & \left.T_{R}=\frac{\alpha L}{2}{\overset{•}{g}}^{\mu \nu }\left(s\right){\overset{•}{g}}_{\mu \nu }\left(s\right)=\frac{1}{2\alpha L}\pi^{\mu \nu }\pi_{\mu \nu }\right., \end{array}$$

(48) $$\begin{array}{ccc} & & \left.V_{o}\left(g,\hat{g},s\right)=h\alpha L\left(g^{\mu \nu }{\hat{R}}_{\mu \nu }-2\Lambda_{c}\right)\right., \end{array}$$

(49) $$\begin{array}{ccc} & & \left.V_{F}\left(g,\hat{g},r\left(s\right),s\right)=\alpha L\kappa g^{\mu \nu }{\hat{T}}_{\mu \nu }\right., \end{array}$$

where $\kappa =8\pi G/c^{2}$, with *G* being the gravitational constant, α, and *L* are dimensional constant 4-scalars, according to the treatment given in Ref. [31], $\Lambda_{c}$ is the cosmological constant and $h=h(g,\hat{g},g_{extr})$ is the variational coefficient, which can be identified with

(50) $$h\left(g,\hat{g}\right)=2-\frac{1}{4}g^{\mu \nu }g_{\mu \nu }.$$

Thus, upon setting the initial condition

(51) $$x_{R}\left(s_{o}\right)=\left\{g_{extr}\left(s_{o}\right)=\hat{g}\left(s_{o}\right),\pi_{extr}\left(s_{o}\right)=0\right\},$$

one finds out that Equations (41) and (42) yield identically

(52) $${\left.\frac{\partial V}{\partial g_{\mu \nu }}\right.}_{g\left(s\right)=\hat{g}\left(s\right)}=0,$$

which in vacuum recovers exactly EFE, namely

(53) $${\hat{R}}_{\mu \nu }\left(s\right)-\frac{1}{2}\hat{R}\left(s\right){\hat{g}}_{\mu \nu }\left(s\right)+\Lambda_{c}{\hat{g}}_{\mu \nu }\left(s\right)=0.$$

Instead, invoking more generally the initial condition

(54) $$x_{R}\left(s_{o}\right)=\left\{g_{extr}\left(s_{o}\right)\neq \hat{g}\left(s_{o}\right),\pi_{extr}\left(s_{o}\right)=0\right\},$$

one finds that

(55) $$\left\{\begin{array}{c} \frac{d}{ds}g_{\mu \nu }\left(s\right)=\frac{1}{\alpha L}\pi^{\mu \nu }\left(s\right),\\ \frac{d}{ds}\pi^{\mu \nu }\left(s\right)=\frac{\partial H_{R}}{\partial g_{\mu \nu }}=h\left(g\left(s\right),\hat{g}\left(s\right)\right)\alpha L{\hat{R}}_{\mu \nu }-\\ \frac{1}{2}\alpha Lg_{\mu \nu }\left(g^{\alpha \beta }{\hat{R}}_{\alpha \beta }-2\Lambda_{c}\right). \end{array}\right.$$

Equations (41) and (42), together with (55), display the classical covariant Hamiltonian structure of GR, i.e., in terms of ordinary differential equations that are expressed in Hamiltonian form. This is a consequence of the functional setting ${\left\{g,\pi \right\}}_{U}$ represented by Equation (38). The basic implication is that standard canonical quantization methods can be adopted [4].

In particular, quantization of the covariant Hamiltonian structure of GR $\left\{x_{R},H_{R}\right\}$ (denoted as g−*quantization*) can be achieved in terms of the Hamilton–Jacobi quantization [31], namely by introducing the quantum correspondence principle

(56) $$\begin{array}{ccc} \left\{x_{R},H_{R}\right\} & \Rightarrow & \left\{x_{R}^{\left(q\right)},H_{R}^{\left(q\right)}\right\}, \end{array}$$

(57) $$\begin{array}{ccc} S(x_{R}) & \Rightarrow & \psi (x_{R}^{\left(q\right)}), \end{array}$$

where $S(x_{R})$ and $\psi (x_{R}^{\left(q\right)})$ are, respectively, the classical Hamilton function associated with $\left\{x_{R},H_{R}\right\}$ and the quantum wave-function which characterizes the corresponding quantum Hamiltonian structure $\left\{x_{R}^{\left(q\right)},H_{R}^{\left(q\right)}\right\}$. Thus, in term of the s—parametrization one finds

(58) $$\begin{array}{ccc} & & \left.\left\{\begin{array}{c} g_{\mu \nu }\left(s\right)\\ \pi^{\mu \nu }\left(s\right)\\ H_{R}\left(g,\hat{g}\left(r\right),\pi ,r,s\right)\\ S(s) \end{array}\right.\Rightarrow \right.\\ & & \left\{\begin{array}{c} g_{\mu \nu }^{\left(q\right)}\left(s\right)=g_{\mu \nu }\left(s\right)\\ \pi^{(q)\mu \nu }\left(s\right)=-i\hbar \frac{\partial }{\partial g_{\mu \nu }\left(s\right)}\\ H_{R}^{\left(q\right)}\left(g,\hat{g}\left(r\right),\pi^{\left(q\right)},r,s\right)=H_{R}\left(g,\hat{g}\left(r\right),\pi^{\left(q\right)},r,s\right)\\ \psi (s) \end{array}\right. \end{array}$$

where $H_{R}^{\left(q\right)}\left(g,\hat{g}\left(r\right),\pi^{\left(q\right)},r,s\right)$ is obtained by replacing $\pi^{\mu \nu }\left(s\right)$ with $\pi^{(q)\mu \nu }\left(s\right)$ in the classical Hamiltonian density $H_{R}\left(g,\hat{g}\left(r\right),\pi ,r,s\right)$, while $x_{R}\left(s\right)=\left\{g^{\mu \nu }\left(s\right),\pi_{\mu \nu }\left(s\right)\right\}$ and $x_{R}^{\left(q\right)}\left(s\right)=\left\{g^{\mu \nu }\left(s\right),\pi_{\mu \nu }^{\left(q\right)}\left(s\right)\right\}$, respectively, denote the corresponding classical and quantum canonical states. Then it follows that the classical Hamilton–Jacobi equation for S(s) is necessarily mapped into a quantum-wave equation (CQG quantum-wave equation) for the quantum wave-function ψ(s). This takes the form of an hyperbolic evolution PDE of the type [4,31]

(59) $$i\hbar \frac{d}{ds}\psi \left(s\right)=H_{R}^{\left(q\right)}\psi \left(s\right),$$

with $\psi \left(s\right)=\psi (x_{R}^{\left(q\right)}\left(s\right),\hat{g}\left(r\right),r\left(s\right),s)$ denoting a 4-scalar quantum wave function. Such an equation is the basis for investigation of QG phenomena, such as the estimate of the graviton mass, the quantum origin of the cosmological constant, the discovery of the phenomenon of the stochastic nature of black-holes (BH) event horizons, the possible screening effect of the cosmological constant near BH event horizons, and the regularization of BH singularities [4]. However, from a theoretical perspective, a further notable feature is the discovery that the CQG quantum-wave Equation (59) admits a unique underlying quantum Hamiltonian structure of CQG theory [32]. This feature can be displayed in two steps as follows.

- *FIRST STEP* —By introducing the Madelung exponential representation
  (60) $$\psi \left(s\right)=\sqrt{\rho (s)}\exp\left\{iS(s)/\hbar \right\},$$
  one obtains for the quantum hydrodynamic fields $\left\{\rho (s),S(s)\right\}$, respectively, the quantum continuity and quantum Hamilton-Jacobi equations [4], i.e.,:
  (61) $$\begin{array}{ccc} \frac{d\rho (s)}{ds}+\frac{\partial }{\partial g_{\mu \nu }\left(s\right)}\left(\rho \left(s\right)V_{\mu \nu }\left(s\right)\right) & = & 0, \end{array}$$
  (62) $$\begin{array}{ccc} \frac{d}{ds}S\left(s\right)+H_{eff}^{\left(q\right)} & = & 0. \end{array}$$
- *SECOND STEP* —By introducing the quantum hydrodynamic canonical momentum
  (63) $$\Pi_{\mu \nu }\left(s\right)=\frac{\partial S(s)}{\partial g^{\mu \nu }\left(s\right)}\equiv \alpha LV_{\mu \nu }\left(s\right),$$
  then the quantum Hamilton–Jacobi PDE (62) can be shown to be equivalent to the set of quantum Hamilton equations
  (64) $$\left\{\begin{array}{cc} \frac{d}{ds}g_{\mu \nu }\left(s\right)= & \frac{\partial H_{eff}^{\left(q\right)}}{\partial \Pi^{\mu \nu }\left(s\right)}=\frac{1}{\alpha L}\Pi_{\mu \nu }\left(s\right),\\ \frac{d}{ds}\Pi^{\mu \nu }\left(s\right)= & -\frac{\partial H_{eff}^{\left(q\right)}}{\partial g_{\mu \nu }\left(s\right)}=-\frac{\partial }{\partial g_{\mu \nu }\left(s\right)}\left[V_{QM}\left(s\right)+V\left(s\right)\right], \end{array}\right.$$
  where the definitions of V(s), $H_{eff}^{\left(q\right)}$ and $V_{QM}\left(s\right)$ can be found in Ref. [4]. Therefore, Equation (64) displays the quantum Hamiltonian structure of GR, as it follows in the framework of CQG theory.

As an important remark concerning CQG theory, one can show [4] that the background metric tensor $\hat{g}\left(s\right)$$\equiv \hat{g}\left(r\left(s\right)\right)$ is uniquely prescribed by the same quantum-wave function ψ(s), solution of Equation (59), in terms of the quantum expectation value (33). Then, the reason why (33) holds in the context of CQG theory is that the quantum probability density ρ(s) can be shown to take the Gaussian form [32]

(65) $$\rho \left(s\right)=\rho_{G}\left(\Delta g-\hat{g}\left(s\right)\right)\equiv k\exp\left\{-\frac{{\left(\Delta g-\hat{g}\left(s\right)\right)}^{2}}{r_{th}^{2}}\right\},$$

where the exponential factor reads

$${\left(\Delta g-\hat{g}\left(s\right)\right)}^{2}={\left(\Delta g-\hat{g}\left(s\right)\right)}^{\mu \nu }{\left(\Delta g-\hat{g}\left(s\right)\right)}_{\mu \nu },$$

while *k* and $r_{th}^{2}$ are, respectively, a normalization factor and the dimensionless semi-amplitude width of the Gaussian distribution. From Equation (65), it follows that $\rho_{G}\left(\Delta g-\hat{g}\left(s\right)\right)$ admits the quantum expectation value (33). Regarding, instead, the determination of the background metric tensor $\hat{g}\left(s\right)\equiv \hat{g}\left(r\left(s\right)\right)$ itself, this follows from the solution of the quantum-modified EFE. This important topic has been discussed in detail elsewhere so we omit for brevity its discussion here [32].

Finally, as shown in Refs. [31,32], the CQG theory allows also the explicit covariant representation of generalized Heisenberg inequalities. This feature is briefly recalled below.

### 4.5. Proof of the Local and Covariant Heisenberg GUP for CQG-Theory

In the case of CQG theory (as for standard quantum mechanics) the proof of the Heisenberg GUP is a consequence of the well-known Robertson uncertainty relation

(66) $$\sigma_{A}\sigma_{B}\geq \left|\frac{1}{2i}\langle \langle \psi |\left[A,B\right]\psi \rangle \rangle \right|.$$

Notice that here all quantities, including the standard deviations, are understood as local, i.e., evaluated at the same 4-position r≡r(s) of the spacetime. Here the notation is standard [32]. Thus, $\langle \langle \psi •|•\psi \rangle \rangle$ identifies the scalar product (defined according to Equation (4), where the integration is performed keeping the background metric tensor $\hat{g}\left(r\right)$ constant) on the complex vector space spanned by the variational tensor *g*, and *A* and *B* are two self-adjoint operators acting on the same vector space and are endowed with quantum expectation values

(67) $$\begin{array}{ccc} \langle \psi |A\psi \rangle & = & \bar{A}, \end{array}$$

(68) $$\begin{array}{ccc} \langle \psi |B\psi \rangle & = & \bar{B}, \end{array}$$

with $\left[A,B\right]=AB-BA$ being their commutator, and finally $\sigma_{A}$ and $\sigma_{B}$ are the local standard deviations

(69) $$\begin{array}{ccc} \sigma_{A}^{2} & \equiv & \langle \psi |{\left(A-\bar{A}\right)}^{2}\psi \rangle , \end{array}$$

(70) $$\begin{array}{ccc} \sigma_{B}^{2} & \equiv & \langle \psi |{\left(B-\bar{B}\right)}^{2}\psi \rangle . \end{array}$$

Then, upon identifying the operators *A* and *B* with the tensor components of the quantum canonical state $x_{R}^{\left(q\right)}\left(s\right)=\left\{g^{\mu \nu }\left(s\right),\pi_{\mu \nu }^{\left(q\right)}\left(s\right)\right\}$, namely identifying *A* and *B* with the s—parametrized fields $A=g^{\mu \nu }\left(s\right)$ and $B=\pi_{\mu \nu }^{\left(q\right)}\left(s\right)$**,** and upon noting that the fundamental commutator relations

(71) $$\left[g^{\mu \nu }\left(s\right),\pi_{\alpha \beta }^{\left(q\right)}\left(s\right)\right]=\hbar \delta_{\alpha }^{\mu }\delta_{\beta }^{\nu }$$

necessarily hold, then it follows that the Heisenberg GUP for the conjugate quantum canonical variables $g^{\mu \nu }\left(s\right)$ and $\pi_{\alpha \beta }^{\left(q\right)}\left(s\right)$ take the form

(72) $$\sigma_{g^{\mu \nu }}\left(s\right)\sigma_{\pi_{\alpha \beta }^{\left(q\right)}}\left(s\right)\geq \frac{1}{2}\hbar \delta_{\alpha }^{\mu }\delta_{\beta }^{\nu }.$$

These inequalities identify the *Heisenberg GUP for CQG-theory*. For completeness, it is worth mentioning here that, provided in place of (4) a suitable definition is given for the scalar product [32], and a further Heisenberg inequality can be proved to hold for the extended conjugate canonical variables, i.e., $\left(s,H_{R}^{\left(q\right)}\left(g,\pi^{\left(q\right)},r,s\right)\right)$, with interesting implications regarding the minimum length problem.

Here we stress that, thanks to the fact that the quantum expectation values defined according to Equation (4) are evaluated while keeping the background metric tensor $\hat{g}\left(r\right)$ constant, the following properties are fulfilled by the Heisenberg inequalities (72):

- *Property #1:* The quantum expectation values of the quantum canonical state, i.e., for $g^{\mu \nu }\left(s\right)$ and $\pi_{\mu \nu }^{\left(q\right)}\left(s\right)$, respectively, namely
  (73) $$\begin{array}{ccc} \bar{g^{\mu \nu }\left(s\right)} & = & \langle \psi |g^{\mu \nu }\left(s\right)\psi \rangle , \end{array}$$
  (74) $$\begin{array}{ccc} \bar{\pi_{\mu \nu }^{\left(q\right)}\left(s\right)} & = & \langle \psi |\pi_{\mu \nu }^{\left(q\right)}\left(s\right)\psi \rangle , \end{array}$$
  identify, as the same $g^{\mu \nu }\left(s\right)$ and $\pi_{\mu \nu }^{\left(q\right)}\left(s\right),$ two 4—tensors defined with respect to the spacetime $\left\{Q^{4},\hat{g}\left(r\right)\right\}$. This occurs, of course, because the integration in Equation (4) is carried out at constant $\hat{g}\left(r\right)$.
- *Property #2:* Thanks to Property #1 the definitions of the standard deviations given above (see, e.g., Equations (3) and (69), (70)) make sense, because both $g^{\mu \nu }\left(s\right)$ and $\bar{g^{\mu \nu }\left(s\right)}$ (and similarly $\pi_{\mu \nu }^{\left(q\right)}\left(s\right)$ and $\bar{\pi_{\mu \nu }^{\left(q\right)}\left(s\right)}$) are 4-tensors defined with respect to the a same spacetime $\left\{Q^{4},\hat{g}\left(r\right)\right\}$.
- *Property #3:* It follows that the Heisenberg inequalities (72) expressed in terms of the local standard deviations $\sigma_{g^{\mu \nu }}\left(s\right)$ and $\sigma_{\pi_{\alpha \beta }^{\left(q\right)}}\left(s\right)$ apply to the 4-tensor functions $A=g^{\mu \nu }\left(s\right)$ and $B=\pi_{\mu \nu }^{\left(q\right)}\left(s\right)$, and thus they are expressed in 4-tensor form, so that they are local and covariant by construction.

## 5. Proof of the Physical Realizability Condition

Let us now address Goals #4 and #5 mentioned in the Introduction. This concerns

- The identification of the quantum objectivity criterion to be fulfilled by the quantum standard deviations, which in the context of QGT occur in the Heisenberg uncertainty inequalities.
- The proof that the same Heisenberg uncertainty inequalities are fulfilled identically only in the context of CQG-theory.

For definiteness, let us denote by $\left\{A(g,r)\right\}$ an arbitrary set of quantum observables depending on the variational field g(r) and by $\langle \psi_{a}|\psi_{b}\rangle$ an appropriate scalar product to be defined on the vector space spanned by the variational tensor field g(r) (see above definition (4)). Notice that here g(r) can be identified either with the constrained variational metric tensor of multiverse ADM theory or the unconstrained variational tensor field of CQG theory. In both cases we shall require that the arbitrary function A(g,r)≡A(g(s),r(s)) is a 4-tensor, represented either in terms of its contra- or co-variant components. However, its quantum expectation values, i.e.,

(75) $$\langle \psi |A^{\mu \nu }\left(g\left(s\right),r\left(s\right)\right)\psi \rangle =\bar{A^{\mu \nu }}\left(r\left(s\right)\right)$$

or

(76) $$\langle \psi |A_{\mu \nu }\left(g\left(s\right),r\left(s\right)\right)\psi \rangle =\bar{A_{\mu \nu }}\left(r\left(s\right)\right)$$

do not necessarily represent a 4-tensor. Therefore, in a proper sense, both $\bar{A^{\mu \nu }}\left(r\left(s\right)\right)$ and $\bar{A_{\mu \nu }}\left(r\left(s\right)\right)$ are not observables since their transformation properties with respect to local point transformations are not properly defined. For the same reason it is obvious that the very notion of quantum standard deviation, which is critical for the validity of the Heisenberg GUP, becomes an issue. In fact, if both $\bar{A^{\mu \nu }}\left(r\left(s\right)\right)$ and $\bar{A_{\mu \nu }}\left(r\left(s\right)\right)$ are not 4-tensors for the quantum function A(g,r) the standard deviation cannot be defined.

Based on these premises it is therefore obvious that the validity of the Heisenberg GUP provides by itself a true *physical realizability condition*. Ultimately, this can be translated in the requirement that both the tensor field A(g,r) and its quantum expectation value $\bar{A(g,r})$, represented equivalently in terms of $A^{\mu \nu }$ or $A_{\mu \nu }$, transform according to 4-tensors, which requires that the following identities should hold identically for an arbitrary tensor field A(g,r). Thus again considering the local dependence A(g,r)≡A(g(s),r(s)) we require:

(77) $$\left\{\begin{array}{c} g^{\mu \alpha }\left(s\right)g^{\nu \beta }\left(s\right){\bar{A}}_{\alpha \beta }\left(r\left(s\right)\right)=\\ \langle \psi \left|g^{\mu \alpha }\left(s\right)g^{\nu \beta }\left(s\right)A_{\alpha \beta }\left(g\left(s\right),r\left(s\right)\right)\psi \right.\rangle ,\\ g_{\mu \alpha }\left(s\right)g_{\nu \beta }\left(s\right){\bar{A}}^{\alpha \beta }\left(r\left(s\right)\right)=\\ \langle \psi \left|g_{\mu \alpha }\left(s\right)g_{\nu \beta }\left(s\right)A^{\alpha \beta }\left(g\left(s\right),r\left(s\right)\right)\psi \right.\rangle . \end{array}\right.$$

Due to the arbitrariness of the tensor field A(g,r), however, it is obvious that the identities (77) generally hold only if:

- The metric tensor which raises and lowers the tensor indices coincides with the background metric tensor $\hat{g}\left(r\right)$.
- Both $A_{\alpha \beta }\left(g\left(s\right),r\left(s\right)\right)$ and $A^{\alpha \beta }\left(g\left(s\right),r\left(s\right)\right)$ and, respectively, ${\bar{A}}_{\alpha \beta }\left(r\left(s\right)\right)$ and ${\bar{A}}^{\alpha \beta }\left(r\left(s\right)\right)$ identify the covariant and contravariant components of tensor fields, which are prescribed with respect to the background spacetime $\left\{Q^{4},\hat{g}\left(r\right)\right\}$.

The consequence is therefore that the *physical realizability condition* provided by the Heisenberg GUP or equivalently by Equation (77) holds identically only in the case of the CQG theory, i.e., only in the case of universe theories.

## 6. Conclusions

A basic issue concerning the theory of Quantum Gravity (QG) is about the quantization of the Hamiltonian representation of the Einstein field equations. The rationale at the basis of the present research is that, in quantum gravity theory, two possible representations of quantum spacetime are in principle available, either based on the notions of multiverse or universe, respectively. The crucial difference being related to the fact that in the first case, infinite possible realizations of the spacetime quantum metric tensor are possible. While in the second case only a unique choice is available in terms of a prescribed background metric tensor to be represented by an appropriate quantum expectation value. From the discussion reported here the following conclusions emerge:

- First, conceptual difficulties arise with the interpretation of the variational formulation of ADM theory in terms of partial differential equations, i.e., intrinsically non-Hamiltonian differential equations. This feature can only be avoided by invoking an additional constraint condition, which amounts to treating the three-dimensional Ricci tensor R(3)(h) as constant (see discussion in Section 4.2 above). Such a constraint condition, however, appears unphysical and therefore difficult to be fulfilled in practice.
- Second, as a consequence, difficulties arise regarding the canonical quantization of ADM theory.
- Third, the requirement of validity of the Heisenberg GUP in 4-tensor form is found to represent an effective theoretical divide. In fact, the validity of the same principle determines a physical realizability condition for QG theories, in that only theories based on the universe representation of spacetime are applicable.
- Fourth, the correct QG theories should therefore themselves admit a 4-tensor representation, a fact that also warrants their frame independence, i.e., the validity in arbitrary GR frames.

As shown here, a possible model of quantum gravity theory satisfying these requirements is realized by the theory of manifestly-covariant quantum gravity (CQG-theory) recently achieved. This is rooted in the canonical quantization of the classical Hamiltonian structure underlying general relativity and displayed by means of its covariant Hamiltonian structure. As a plus feature, such an approach also overcomes the non-Hamiltonian feature displayed by the Euler–Lagrange equations that characterize ADM theory.

In conclusion, what emerges is that CQG-theory appears to meet the physical requirement set by the Heisenberg GUP. In fact, as shown here, the theory permits to reach well-defined conclusions regarding the proof of validity of such a principle. Nevertheless, further investigations are demanded to understand the full implications of the present theory, with particular reference to the implications of the background representation of spacetime.

## Data Availability

Data are contained within the article.

## Appendix A. Classical Spacetimes

In this Appendix A we recall the three main classical spacetimes, historically represented, respectively, by the Galileian spacetime structure due originally to Galilei and Newton, the Minkowski spacetime structure formulated in the context of special relativity, and finally, the Lorentzian spacetime structure due to Einstein and arising in GR.

- THE GALILEIAN SPACETIME STRUCTURE. The spacetime, or “cosmo” (also sometimes called extended configuration space), is identified with a so-called “*Galileian spacetime structure*”, namely the four-dimensional spacetime represented by the direct product $A^{4}\equiv A⊗A^{3}$, with *A*, $A^{3}$ and $A^{4}$ being affine spaces on the time axis R, $R^{3}$ and $R^{4}$ the configuration space of a point particle. The first two, *A*, $A^{3}$ are also Euclidean spaces being endowed, respectively, with the following Euclidean distances:
  (a) the first one is identified with the application *absolute time*
  (A1) $$t\left(a-b\right):R^{4}\to R,$$
  with R being the oriented time axis;
  (b) the second one with the Euclidean distance in space
  (A2) $$\rho \left(a,b\right):R^{4}\to R,$$
  defined as
  (A3) $$\rho \left(a,b\right)=\sqrt{\sum_{i=1,3}{\left(r_{a}^{i}-r_{b}^{i}\right)}^{2}},$$
  where $a\equiv (r_{a}^{0},r_{a}^{i}),$$b\equiv (r_{b}^{0},r_{b}^{i})$ are simultaneous events, i.e., such that $t\left(a-b\right)=r_{a}^{0\prime }-r_{b}^{0}=0$, so that by construction the vector a−b belongs to the Euclidean space $R^{3}$.

The second one concerns the cosmological picture, i.e., the model proposed by Newton based on classical mechanics for matter in all its forms (including the formation of arbitrary physical bodies, planets, stars, etc.), which he ascribes uniquely to ensembles of like point particles, whose dynamics should be governed by Newtons equations for all particles.

- NEWTONIAN COSMOLOGY. The Galileian spacetime was assumed by Newton to be filled with an ensemble of (finite or numerable infinite number of) like point particles, namely a so-called *Newtonian N-body system* (whose particles are evenly distributed to form all observed objects, namely the “cosmo”). The Newton’s grand contribution to cosmology, now called Newtonian Cosmology, was that the time evolution of the cosmo should be unique, i.e., *deterministic*. In fact, Newton’s conjecture is that all point particles of the “cosmo” should be governed by a corresponding Newton’s equation.

However both the Galileian structure of spacetime and the deterministic Newtonian cosmology are known to become physically incorrect in certain conditions. For Newtonian cosmology this happens if the point particles are treated as hard smooth spheres that are endowed with an infinitesimal radius. In such a case one can show that the dynamical particle evolution of the Newtonian N-body system becomes stochastic, i.e., *non deterministic* when the multiple particle collisions occur. Similarly the Galileian structure of spacetime fails in the case of special- and general-relativistic Newtonian N-body systems.

- THE MINKOWSKI SPACETIME STRUCTURE. The Minkowski spacetime is represented by the set $\left\{Q^{4},\eta \right\}$ equipped with a (Minkowski) inner product. Here the notations are as follows:
  (a) $Q^{4}$ denotes the space $A^{4}$, i.e., the affine space on $R^{4}$;
  (b) η is a constant and diagonal metric tensor η with signature $\left(-,+,+,+\right)$ and thus defined as
  (A4) $$\eta \equiv \left|\begin{array}{cccc} -1 & 0 & 0 & 0\\ 0 & 1 & 0 & 0\\ 0 & 0 & 1 & 0\\ 0 & 0 & 0 & 1 \end{array}\right|,$$
  (c) the Minkowski inner product is defined as
  (A5) $$s\left(a,b\right)=\sqrt{\eta_{ij}\Delta r^{i}\Delta r^{j}},$$
  where $a=\left(r_{a}^{0},r_{a}^{1},r_{a}^{2}\;,r_{a}^{3}\right)$ and $b=\left(r_{b}^{0},r_{b}^{1},r_{b}^{2}\;,r_{b}^{3}\right)$ are arbitrary points of $Q^{4}$, while $\Delta r^{i}$ for i=0,3 denote the displacements $\Delta r^{i}=$$r_{a}^{i}-r_{b}^{i}$.

Formally similar is the spacetime in general relativity, i.e., in the case of a general curved spacetime. This implies that generally the spacetime metric tensor is non-constant and its form must be prescribed according to the Einstein field equations.

- THE LORENTZIAN SPACETIME STRUCTURE. The Lorentzian spacetime is represented by the set $\left\{Q^{4},g\right\}$ equipped with a (Riemannian) inner product and non-constant symmetric metric tensor g(r). Here the notations are as follows:
  (a) $Q^{4}$ denotes the space $A^{4},$ i.e., the affine space on $R^{4}$;
  (b) g(r) is a symmetric metric tensor with signature $\left(-,+,+,+\right)$, and thus defined generally as
  (A6) $$g\left(r\right)\equiv \left\{g^{\mu \nu }\left(r\right)\right\}\equiv \left\{g_{\mu \nu }\left(r\right)\right\};$$
  (c) The Riemannian inner product is defined as
  (A7) $$ds=\sqrt{g_{ij}\left(r\right)dr^{i}dr^{j}},$$
  where $a=\left(r_{a}^{0},r_{a}^{1},r_{a}^{2}\;,r_{a}^{3}\right)$ and $b=\left(r_{b}^{0},r_{b}^{1},r_{b}^{2}\;,r_{b}^{3}\right)$ are arbitrary points of $Q^{4}$ such that $dr^{i}$ for i=0,3 identify infinitesimal displacements $dr^{i}=$$r_{a}^{i}-r_{b}^{i}$.
